# Dosimetric Characterization of DSF/NaOH/IA-PAE/*R.* spp. Phantom Material for Radiation Therapy

**DOI:** 10.3390/polym15010244

**Published:** 2023-01-03

**Authors:** Damilola Oluwafemi Samson, Ahmad Shukri, Nurul Ab. Aziz Hashikin, Siti Hajar Zuber, Mohd Zahri Abdul Aziz, Rokiah Hashim, Mohd Fahmi Mohd Yusof, Nor Ain Rabaiee, Sylvester Jande Gemanam

**Affiliations:** 1 School of Physics, University Sains Malaysia, Penang 11800, Malaysia; 2 Department of Physics, Faculty of Science, University of Abuja, Abuja 900211, Nigeria; 3 Advanced Medical and Dental Institute, University Sains Malaysia, Penang 13200, Malaysia; 4 School of Industrial Technology, University Sains Malaysia, Penang 11800, Malaysia; 5 School of Health Sciences, University Sains Malaysia, Kota Bharu 16150, Malaysia; 6 Department of Radiology, Kulliyyah of Medicine, International Islamic University, Kuantan 25200, Malaysia; 7 Department of Physics, Benue State University, Makurdi 102119, Nigeria

**Keywords:** dosimetric properties, tissue-equivalent phantom, absorbed dose, IA-PAE, radiation therapy

## Abstract

**Background:** Different compositions of DSF/NaOH/IA-PAE/*R.* spp. composite particleboard phantoms were constructed. **Methods:** Photon attenuation characteristics were ascertained using gamma rays from ^137^Cs and ^60^Co. Absorbed doses at the location of an ionization chamber and Gafchromic EBT3 radiochromic films were calculated for high-energy photons (6 and 10 MV) and electrons (6, 9, 12, and 15 MeV). **Results:** The calculated TPR_20,10_ values indicate that the percentage discrepancy for 6 and 10 MV was in the range of 0.29–0.72% and 0.26–0.65%. It was also found that the relative difference in the $d_{max}$ to water and solid water phantoms was between 1.08–1.28% and 5.42–6.70%. The discrepancies in the determination of PDD curves with 6, 9, 12, and 15 MeV, and those of water and solid water phantoms, ranged from 2.40–4.84%. Comparable results were found using the EBT3 films with variations of 2.0–7.0% for 6 and 10 MV photons. Likewise, the discrepancies for 6, 9, 12, and 15 MeV electrons were within an acceptable range of 2.0–4.5%. **Conclusions:** On the basis of these findings, the DSF/NaOH/IA-PAE/*R.* spp. particleboard phantoms with 15 wt% IA-PAE addition level can be effectively used as alternative tissue-equivalent phantom material for radiation therapy applications.

## 1. Introduction

Phantoms have become essential for quality assurance (QA) and quality control (QC) in a variety of medical procedures involving radiation. The earliest phantoms consisted of water or wax, but wax phantoms had a number of issues. Wax formulations differed greatly depending on the type of wax used and, at low energies, deviated from tissue equivalence [1]. On the other hand, water has been described as the standard and most universal phantom material for dosimetry measurements of photon and electron beams. As the use of liquid water can prove to be challenging and inconvenient in certain situations, because of its surface tension and the uncertainty in positioning the detector near the surface, solid homogeneous phantom materials have achieved substantial recognition [2]. The benefit of these phantoms allows the measurement of the interaction of ionizing radiation in the human body, which enables the range of doses in various organs and tissues to be measured according to their sensitivity. The most widely used tissue-equivalent material (TEM) are those that are both easy to work with and relatively inexpensive. The usage of natural, readily available, and cheap phantom material, such as wood (*Rhizophora* spp.), is often of interest.

*Rhizophora* spp. (*R.* spp.) has received increasing attention for industrial applications due to its fast-growing nature, high productivity, quick maturity, and high strength, with advancement in processing technology and increased market demand. The chemical composition of *R.* spp. is very similar to those of TEM normally utilized as phantoms for radiation therapy when compared with other wood species [3,4,5,6,7,8]. Moreover, *R.* spp. possesses convenient morphological characteristics and physiological adaptations, with moisture content ranging from 5–10% and basic physical density within 0.90–1.04 gcm^−3^ [9]. Various researchers have shown that *R.* spp. is a highly attractive material for use as an effective TEM for a wide range of benefits, including high-energy photon and electron radiation therapy, as well as X-ray imaging [5,6,7,10,11,12]. However, due to its shortcomings, such as the tendency to be warped, cracked, degraded, and weakened over time, the usage of appropriate resins with unique characteristics in the development of *R.* spp. particleboards has been reported [5,6,7,8,11,12]. The type of these curing resins and their chemical properties are also crucial criteria that should be well-decided for particleboard phantom formation and structure.

Among the various forms of modifying resins, synthetically-based ones are the most commonly adopted [13], but prolonged human exposure to non-renewable resources has been shown to cause chronic toxicity, myeloid leukemia mortality, and lymphohematopoietic malignancies [14,15,16]. In relation to TEM studies, synthetically based resins were also found not to be compatible with the intended density and radiation attenuation properties (RAPs) of *R.* spp. particleboards as compared to water [17]. On the other hand, bio-based materials, such as soy protein (DSF—defatted soy flour) developed in wood resin, have been validated through specific independent studies because of their ready availability and low cost, coupled with the fact that they are biodegradable, biocompatible, and eco-friendly [6,7,15,16,18,19,20].

DSF is a highly oxygenated carbon compound, which makes it attractive for use in the development of phantom materials equivalent to tissue and water, and it can be appropriate either as an uncured or a cured bio-based adhesive [18]. Uncured DSF was, however, identified as a weak adhesive, and a chemical change is needed to break the internal bonds and disperse the polar protein molecules [6,16,18,19,20,21,22,23,24]. The most commonly used cross-linking agents for DSF are itaconic acid polyamidoamine-epichlorohydrin (IA-PAE), epoxy, formaldehyde, glutaraldehyde, and glyoxal. Since some of these curing agents also have a deleterious environmental impact, as well as being non-renewable, IA-PAE has been considered as an alternative cross-linker for DSF-based adhesives [6,18,19,20,24]. The cross-linking reaction of DSF with NaOH/IA-PAE resins is highly regarded for their incomparable multifunctionality, enhanced physical and mechanical characteristics, stable water resistance, and good wood-bonding ability [6].

The current study aims to construct and examine the dosimetric characterization of bio-based particleboard phantoms for radiation therapy by integrating DSF-based resins—*R.* spp. particles of size ≤74 μm, NaOH (10 wt%)—and four different treatment levels of IA-PAE (0, 5, 10, and 15 wt%). High energy photon attenuation measurements were ascertained using a Ludlum setup with ^137^Cs and ^60^Co sources with effective photon energies of 0.662 and 1.250 MeV. A linear accelerator (LINAC) was utilized to determine the dosimetric characteristics of the DSF/NaOH/IA-PAE/*R.* spp. particleboard phantoms. This is done using a cylindrical Farmer-type ionization chamber (IC) (NE 2581/334) and Gafchromic EBT3 radiochromic films to evaluate the tissue–phantom ratio (TPR_20,10_), percentage depth dose (PDD) and beam profile of the samples for high energy photon (6 and 10 MV) and electron (6, 9, 12 and 15 MeV) beams. The findings were compared with those of appropriate standard phantom materials (water and solid water) utilized in radiation therapy.

## 2. Materials and Methods

### 2.1. Preparation of Bio-Based Adhesives

As previously reported [6,7], the synthesized IA-PAE solution had a solid content of 55.96 ± 0.01 wt%, a pH of 6.68 at 27.58 °C, and an apparent viscosity of 100.40 ± 0.25 mPa.s, comparable to commercial PAE-soy protein (C-PAE-SF) and IA-PAE reported by Gui et al. [19]. The DSF-based bio-adhesives were prepared at room temperature by dissolving 35 g of DSF under steady mechanical stirring in distilled water (65, 50, 45, and 40 g) for 0.5 h following the procedure described by Samson et al. [6]. Various concentrations of the prepared IA-PAE (0, 5, 10 and 15 wt%) were then applied to the uniform mixtures and moderately stirred for 0.5 h. The cured DSF/IA-PAE slurry mixture was maintained at pH 11.0 with 2N of NaOH (10 wt%) solution, since pH 11.0 is the optimum condition for cross-linking reactions [25].

### 2.2. Fabrication of DSF/NaOH/IA-PAE/R. spp. Particleboard Phantoms

At the start of the process, all sample formulations with particle size ≤ 74 μm were thoroughly mixed by hand as they were applied for 0.5 h to the DSF/NaOH/*R.* spp. mixture with different IA-PAE content. Thereafter, they were mixed evenly for another 10 min using a rotary mixer machine to ensure the uniformity of the samples. By using a mould of dimension (30 cm × 30 cm × 1.0 cm), the mixtures were subsequently cold-compressed for 10 min using a hydraulic press machine (0.49 MPa, 5 min, and 0 °C) fitted with stops to achieve a target density of 1.0 gcm^−3^ at room temperature and relative humidity of 55%. The stacked mats were then constructed using a hydraulic hot press machine at 170 °C for 20 min with 20 MPa [6]. A total of 150 units of DSF/NaOH/IA-PAE/*R.* spp. particleboard phantoms were developed. Table 1 and Table 2 have been tabulated to examine the physico-mechanical and dimensional stability properties (PMDSP) (MC—moisture content, SC—solid content, IB—internal bonding, MOR—modulus of rupture, MOE—modulus of elasticity, TS—thickness swelling, and WA—water absorption), elemental compositions, effective atomic numbers ($Z_{eff}$), and electron densities ($N_{el}$) of the particleboards and standard phantom materials. As can be seen, Table 1 shows that sample A_15_ with 15 wt% IA-PAE concentrations provides the ascribable parameters and meets the minimum requirements of Type 8, Type 13, and Type 18, according to JIS A-5908 [26]. The $Z_{eff}$ and $N_{el}$ of these phantoms, which were the exclusive parameters used to characterize various types of materials, were found to be comparable to those of water and other commercial phantom materials (Table 2).

Using the gravimetric technique (Equation (1)), the average particleboard densities (ρ) were assessed, and the propagation of uncertainty was deduced based on the external dimensions using Equation (2):

(1) $$\rho =\frac{m}{l\times w\times h}$$

(2) $$d\rho =\left(\frac{dm}{m}+\frac{dl}{l}+\frac{dw}{w}+\frac{dh}{h}\right)\rho \;$$

where, m, l, w, and h denote the respective particleboard mass, length, width, and thickness; dm, dl, dw, and dh are the uncertainties in m, l, w, and h, respectively.

The computed tomography (CT) image modality was achieved based on a previous technique detailed by Samson et al. [7]. The parameters of the various standard phantom materials, compared with DSF/NaOH/IA-PAE/*R.* spp., are listed in Table 3. The results of the average density show that the density of A_15_ is within the range found for water and other commercial phantom materials. According to the results, the mean HU values and ED of the A_15_ were near to those acceptable standard reference equivalent materials [7], while a significant variation is observed with A_o_, A_5_, and A_10_, respectively. This might explain the fact that better attenuation abilities were observed when X-ray beams passed through the corresponding sample (A_15_). Therefore, the A_15_ sample formulation showed the potential to replicate human tissue because it has a comparable dynamic and is higher in terms of stability as a medical phantom.

### 2.3. Measurement of RAPs

**Figure 1:**
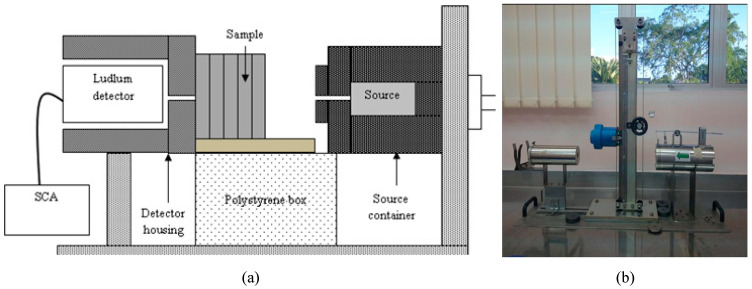
Ludlum setup: (**a**) schematic diagram and (**b**) actual experimental setup.

The attenuation properties were determined using a Ludlum lead equivalent setup, as depicted in Figure 1. ^137^Cs and ^60^Co sealed sources with effective gamma energies of 0.662 and 1.250 MeV were used to provide the incident photons. The sources and the Ludlum NaI(TI) detector, with diameters of 2.5 cm and 6.5 cm, were encapsulated in a lead container with collimation of diameter 0.5 cm and thickness of 2 cm to simulate the line source projection and avoid leakage. An aluminum (Al) plate of dimension 7 cm × 7 cm, with an approximate thickness of 0.1 cm, was used as an attenuator to produce the scattered photons. The optimum distance between the source compartment and the Al plate and between the Al plate and the detector compartment was 30 cm, whereas the distance between the phantom samples and the detector compartment was 6.2 cm. The transmitted photons from the source were collected and detected using the Ludlum scintillation detector connected to a single channel analyzer (SCA).

The linear attenuation coefficient (LAC) and mass attenuation coefficient (MAC) are the fundamental parameters to evaluate the dosimetric and radiation shielding performance of any composite material. These commonly used parameters provide some information on the possibility of photon interaction processes with matter per unit thickness. As a photon beam propagates through a homogeneous medium, the beam intensity at depth t is assigned as $I_{t}$, whereas the beam intensity at a reference point in the absorbing material (t=0), is assigned as $I_{o}$. This can be described by the familiar Beer-Lambert’s law (Equation (3)) [6,7].

(3) $$\mu =\frac{1}{x}\ln\left(\frac{I_{o}}{I_{t}}\right)$$

(4) $$\mu_{m}=\frac{\mu }{\rho }=\frac{1}{\rho t}\ln\left(\frac{I_{o}}{I_{t}}\right)=\frac{A}{M}\ln\left(\frac{1}{T}\right)$$

where μ (cm^−1^) denotes the LAC, ρ (g·cm^−3^) is the density, x (cm) and t (g·cm^−2^) are the physical thickness and mass thickness (mass per unit area), *T* is the transmittance, *M* (g) is the mass of the sample material, *A* is the cross-sectional area (cm^2^), and $\mu_{m}$ (cm^2^g^−1^) indicates the total MAC. The total $\mu_{m}$ values were calculated on the basis of the mixture rule by using the weight fraction ($\omega_{i}$) for each element *i* of the particleboard materials, as expressed in Equations (5) and (6):

(5) $$\mu_{m}={\left(\frac{\mu }{\rho }\right)}_{DSF-based\;particleboard}=\omega_{1}{\left(\frac{\mu }{\rho }\right)}_{1}+\omega_{2}{\left(\frac{\mu }{\rho }\right)}_{2}+\ldots =\overset{N}{\underset{i=1}{\sum }}\omega_{i}{\left(\frac{\mu }{\rho }\right)}_{i}$$

(6) $$\omega_{i}=\frac{n_{i}A_{i}}{\sum_{i}n_{i}A_{i}}=\frac{{\tilde{\rho }}_{i}}{\rho }\;$$

where $\;n_{i}$ denote the number of atoms of the i^th^ individual element, $A_{i}$ is the atomic weight, and ${\tilde{\rho }}_{i}$ is the actual mass density. The related cumulative discrepancies in the experimental MAC were obtained by using the propagation of error relationship from ambiguities in $I_{o},I_{t},\;x$ and areal density (ρ) [7]:

(7) $$∆\left(\frac{\mu }{\rho }\right)=\frac{1}{\rho }\sqrt{\left[{\left(\frac{∆I_{t}}{I_{t}}\right)}^{2}+{\left(\frac{∆I_{o}}{I_{o}}\right)}^{2}+{\left(\ln\frac{∆I_{o}}{I_{t}}\right)}^{2}+{\left(\frac{∆x}{x}\right)}^{2}\right]}$$

where $∆I_{t},\;∆I_{o}$, and ∆x are the errors in the intensities $I_{t},\;I_{o}$, and thickness x of the sample material, respectively. Paired *t*-test using SPSS (V22.0) was used to calculate any variations in $\mu_{m}$ values, as compared with the value of water ascertained via the photon cross-section database (XCOM) [29]. The half-value layer (HVL–$X_{1/2}$) is used to assess how far X-ray penetrates the particleboard samples, which were used to verify the performance of the patient’s radiation exposure. It can be defined, as given in Equation (8), whereas Equation (9) is the relationship between the mean free path $\left(\mathrm{MFP}-\lambda \right)$ and $X_{1/2}$ [7,8].

(8) $$\mathrm{HVL},\;X_{1/2}=\frac{0.693}{\mu_{m}\times \rho }$$

(9) $$\mathrm{MFP},\;\lambda =\frac{X_{1/2}}{0.693}$$

### 2.4. Dosimetric Evaluation of DSF/NaOH/IA-PAE/R. spp. Particleboard Phantoms

Samples with up to 15 wt% IA-PAE addition were selected because of their optimum characteristics, and a total of 34 units of DSF/NaOH/IA-PAE/*R.* spp. particleboard phantoms of sizes 30 cm × 30 cm × 1.0 cm and 30 cm × 30 cm × 0.5 cm, simulating the dimensions of widely used solid water phantom slabs (CIRS Inc., Norfolk, VA, USA), were fabricated. Additionally, two of these slabs were designed with slots to accommodate the cylindrical IC. The Farmer-type IC was used due to its unique features, such as high precision, stability, dose rate independence, excellent linearity, little to no fading, and equivalency to soft tissue nature. All experimental measurements with both photon and electron beams were carried out on the medical Elekta Synergy PRIMUS LINAC at the Department of Oncological and Radiological Science, Advanced Medical and Dental Institute, Universiti Sains Malaysia (USM).

### 2.5. Determination of Photon Beam Quality Index (TPR_20,10_—Tissue Phantom Ratio)

**Figure 2:**
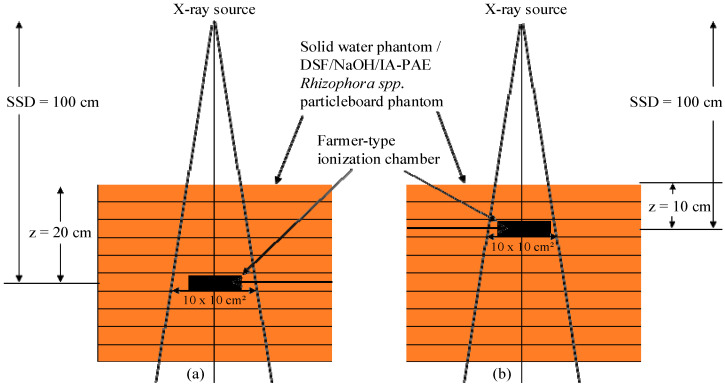
Measurement setup for TPR_20,10_: (**a**) depths z = 20 cm and (**b**) depth z = 10 cm below the water surface at field size (10 cm × 10 cm) and SSD (100 cm).

Samples of DSF-based particleboard and solid water phantoms were mounted and aligned on the central axis of the beam, followed by the insertion of the IC into an electrometer (Model PTW-Unidos ET10008/081134) at depths z = 20 cm and z = 10 cm below the water surface at 10 cm × 10 cm field size and 100 cm SSD, as depicted in Figure 2. Before taking any reading, the IC and electrometer were warmed up for 10 min. For each of the phantom samples, three exposures were made at the two depths, and the average charge collected was evaluated. The expression related to the charge collected at the two depths can be expressed as:

(10) $$TPR_{20,10}=\frac{Q_{20}}{Q_{10}}$$

where $Q_{20}$ and $Q_{10}$ are the respective charge (nC) collected at depths z = 20 cm and z = 10 cm for DSF-based particleboards, water, and solid water phantoms, respectively.

### 2.6. PDD Evaluation Using IC

Samples of 15 cm thickness were placed on the LINAC couch to establish the photon and electron beams with backscatter. The calibrated IC with an inner volume of 0.6 cm^3^, connected to the electrometer, was placed in the chamber slot to acquire PDDs in the DSF-based particleboards, water, and solid water phantoms, as displayed in Figure 2. The slabs of solid water were selected for the phantom material, as it was found to be appropriate for dosimetry of high energy photon and electron beams [30]. The IC and phantoms were positioned in an isocentre distance of the LINAC at an SSD of 100 cm using the front pointer device, and the field size was set at 10 cm x 10 cm on the surface in accordance with the calibration parameters in the dosimetry protocol of IAEA TRS-398:2000 [31]. Exposures were rendered using photon beams of 6 and 10 MV and electron beams of 6, 9, 12, and 15 MeV with 100 monitor units (MU). Particleboard slabs were added above the IC to assess the ionization at depth below the surface of the phantoms, and the SSD and field size were subsequently readjusted. During these measurements, both the gantry and collimator angles were set to zero degrees. The PDDs were measured from the phantom surface at 0 cm until a depth of approximately 20 cm was reached along the central axis, with a measurement interval of 1 mm from the surface to 2 cm depth followed by 2.5 cm and then 3 cm up to 25 cm. The PDD determination for each depth took 6 s. Exposure using electron beams was achieved by adopting an applicator to the LINAC treatment head. After each exposure, a time delay of 120 s was applied before the next phantom slab was inserted in order to take proton production into account. The PDD values were expressed as a percentage of the absorbed dose at a given depth D′ to the absorbed dose at a specified reference depth (maximum depth) D″ along the central axis of the phantom samples (Equation (11)). The discrepancy in the calculated PDD was estimated as a percentage (D%), as given in Equation (12):

(11) $$PDD=\frac{D^{\prime }}{D^{\prime \prime }}\times 100\%\;$$

(12) $$D\left(\%\right)=\frac{PDD_{\left(DSF\right)}-PDD_{\left(water/solid\;water\right)}}{PDD_{\left(water/solid\;water\right)}}\times 100\;$$

where $\;PDD_{\left(DSF\right)}$ is the PDD for the constructed DSF-based *R.* spp. particleboard phantom samples and $PDD_{\left(water/solid\;water\right)}$ is the PDD for the water and solid water phantoms. The PDD curves were plotted for 6 and 10 MV photons, as well as for 6, 9, 12, and 15 MeV electrons.

### 2.7. PDD Evaluation Using Gafchromic EBT3 Radiochromic Films

**Figure 3:**
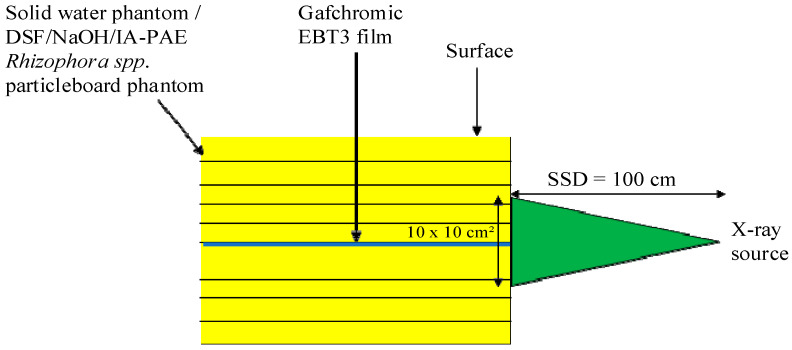
Experimental setup used for PDD evaluation.

The Gafchromic EBT3 radiochromic film sheets (Lot #: 05161903), with dimensions of 20.3 cm × 25.4 cm, were inserted between the DSF-based particleboard phantoms and solid water phantoms in a portrait orientation due to their near tissue- and water-equivalent characteristics, and 10 cm of phantom material was placed under the film to ensure sufficient backscatter. Phantom slabs were inserted above the film, and the SSD and field size were readjusted afterward. The measurements were repeated until a depth of almost 20 cm, and the results were compared with that of water and solid water phantoms. All films were marked with reference points to indicate the film orientations relative to the gantry. Irradiation was made parallel to the beam for a static 10 × 10 cm^2^ field size at 100 cm SSD and with a dose ranging from 0 to 700 cGy. Three films were exposed for each photon and electron energy. The irradiated films were kept at room temperature for 24 hrs post-irradiation to allow time for the polymerization reactions in the film to stabilize and produce a stable optical density measurement [32]. The films were then processed with an EPSON Expression 10,000 XL flatbed scanner. To acquire images, a desktop computer was interfaced with the scanner, and VeriSoft^®^ software 5.1 was used for image scanning and capture. The experimental setup for the PDD evaluation is highlighted in Figure 3. The PDD data were normalized to the maximum dose, expressed as a percentage, and the percentage variation was measured, as indicated in Equations (11) and (12).

## 3. Results and Discussion

### 3.1. Density Measurement of DSF-Based R. spp. Particleboard Phantoms

**Figure 4:**
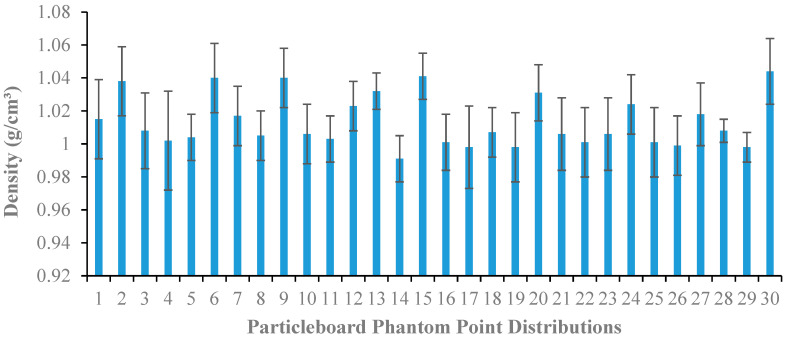
Average density with point distributions of DSF/NaOH/IA-PAE/*R.* spp. composite particleboard phantoms.

Figure 4 displays the variation of average densities with point distribution of DSF-based *R.* spp. composite particleboard phantom slabs. It is seen that the constructed particleboard phantoms exhibit acceptable quality values of average densities to those of water (1.00 gcm^−3^) and solid water phantom (1.04 gcm^−3^) in the range between 0.99 ± 0.01–1.04 ± 0.03 gcm^−3^, making them potentially suitable for use in the fabrication of tissue-equivalent phantom materials. This is attributed to better adhesive-coated particles that provide intimate contact with the mat’s wood particles and, thus, increase the bonding capabilities of the particleboards. This revealed that the combination of DSF/NaOH/IA-PAE with an increased percentage concentration of IA-PAE up to 15 wt% leads to an improvement in the average mass density of the particleboards approaching the value of water. These findings are in good agreement with previous studies of the average density of particleboard phantoms for dosimetric applications at high photon and electron energies utilized in radiation therapy [5,10].

### 3.2. Evaluation of RAPs

**Figure 5:**
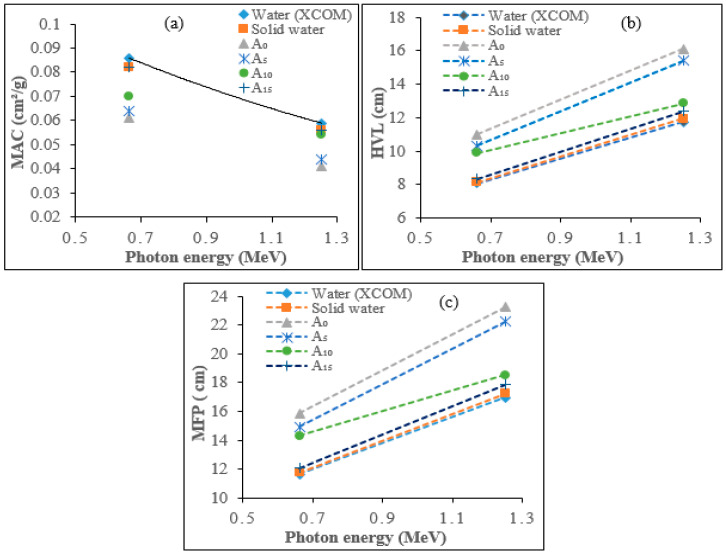
RAPs of DSF-based composite phantoms, water (XCOM), and solid water phantom against gamma energies: (**a**) MAC, (**b**) HVL, and (**c**) MFP.

The experimental computation has been performed in order to obtain the total LAC and MAC values for photon energies of 0.662 and 1.250 MeV and compared with those of solid water phantom and theoretical values of water using cross-section data (XCOM), as displayed in Table 4. The errors in density, thickness, incident, and transmitted gamma-ray intensities were used to evaluate the uncertainties in experimental MAC. The dependency of MAC values on photon energies can be explained by the dominance of partial photon interactions (e.g., photoelectric absorption, coherent scattering, incoherent scattering, and pair production) with the samples. As we know, the photoelectric effect dominates below, and pair production dominates above 1 MeV, whilst Compton scattering dominates at around 1 MeV [8,33]. The calculated values of LAC ranged from 0.059–0.083 cm^−1^ for 0.662 MeV photon energy, while for 1.250 MeV, the observed LAC values were within 0.043–0.056 cm^−1^. Additionally, the observed total MAC values for these photon energies ranged between 0.059–0.082 cm^2^g^−1^ at 0.662 MeV, whereas for 1.250 MeV, the MAC values were found to range between 0.041–0.056 cm^2^g^−1^. The estimated errors in experimental total MAC values for all the samples were less than 0.028%. As observed from Figure 5a, the difference of total MAC values with the incident photon energy for all composite particleboard samples and those of solid water phantom and water (XCOM) is almost identical as IA-PAE concentration increases with A_15_, depicting higher MAC values for both photon energies, potentially providing a useful approximation of tissue-equivalent phantom materials. As expected, by increasing the incident photon energy, the total MAC values in all samples decreased slightly. This behavior may be due to the incoherent scattering process, which becomes the dominant mechanism in this region [34]. This can be ascribed to the fact that the Compton scattering cross-section is inversely proportional to the incoming photon energy (E^−1^) and varies linearly with atomic number. In all the investigated samples, by increasing the incident photon energy, the highest HVL and MFP values were found for samples containing A_0_ and A_5_, while the lowest values were found for water (XCOM) and solid water phantom (Figure 5b,c). It was also observed that, in all samples and for all energies, A_15_ has the lowest HVL and MFP values with approximately no noticeable difference relative to those of solid water phantom and water (XCOM), which implies a higher radiation absorption ability. A comparison between the calculated values shows reasonable agreement with 15 wt% IA-PAE, solid water phantom, and theoretical values of water (XCOM), as depicted by the $\chi^{2}$ values (Table 5). It can be seen that, among the selected samples, A_15_ provided the least values of $\chi^{2}$ (0.044). This revealed, with an insignificant difference, the closest value of RAPs to those of solid water phantom and the theoretical value for water (XCOM).

### 3.3. Dosimetric Characteristics of DSF-Based R. spp. Particleboard Phantoms

#### 3.3.1. Measurement of Photon Beam Quality Index

The tissue-phantom ratio (TPR_20,10_) remains the most appropriate parameter for ascertaining the beam quality of a clinical photon beam. It is believed that material with near TPR_20,10_ to water has similar RAPs to those of water and soft tissue [35]. The measured TPR_20,10_ values of DSF-based *R.* spp. particleboards (sample A_15_), solid water, and water phantoms for 6 and 10 MV photon beams with the use of IC are presented in Table 6 and Table 7. The result indicates that the percentage discrepancies of sample A_15_ in comparison to those of solid water and water phantoms are in the range between 0.29–0.72% for 6 MV photons. Likewise, the discrepancies for the 10 MV photon beam are within the acceptable range of 0.26–0.65%. These results are in good agreement with previous work on the TPR_20,10_ of renewable resources in the respective photon energy ranges [5,10].

Sample A_15_ with *p*-values of 0.071 and 0.069 for 6 and 10 MV photons showed no significant difference to those of water and solid water phantoms in the photon beam radiation quality, as presented in Table 8 and Table 9. These findings demonstrated that DSF/NaOH-based *R.* spp. particleboard phantoms with 15 wt% IA-PAE (sample A_15_) provide the ascribable characteristics that are proper as appropriate tissue-equivalent phantom materials.

#### 3.3.2. Determination of PDD Photon Beams Using IC

**Figure 6:**
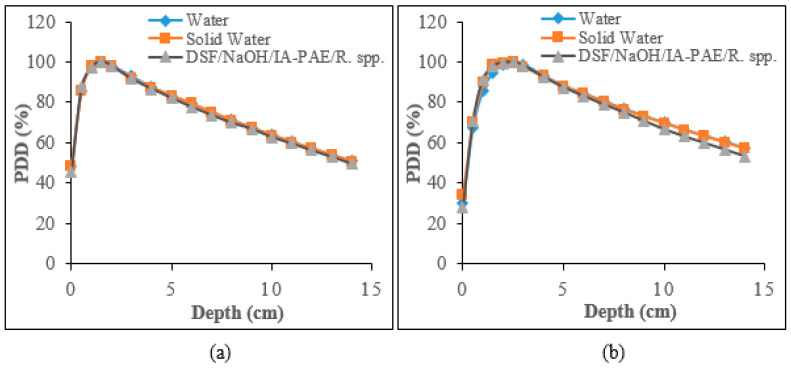
PDD curves for water, solid water, and DSF/NaOH/IA-PAE/*R.* spp. particleboard phantoms using IC for: (**a**) 6 MV and (**b**) 10 MV photons.

The measured PDD values of 6 and 10 MV photon beams with the use of IC for sample A_15_, water, and solid water phantoms are shown in Figure 6. The computed profiles were normalized to the maximum dose in the depth-dose profile positioned symmetrically opposite the IC within the photon beam to ensure that the profiles being compared were identical to those of water and solid water phantoms. The dose first increases steadily below the surface dose ($d_{s}$), reaches a maximum value ($d_{max}$) at $z_{max}$, then decreases almost gradually until it reaches $d_{ext}$ at the patient’s exit position. The discrepancies in the d_max in comparison to those of water and solid water phantoms were at most 1.08% and 1.28% for 6 MV photons (Figure 6a). On the other hand, for 10 MV photons (Figure 6b), the observed percentage differences in the $d_{max}$ were found to be 5.42% and 6.70% at the dose build-up region, which is the region from the phantom surface to the depth at d_max and the equilibrium region. The greatest difference was recorded for 10 MV photons for all the phantoms, which is similar to previous observations by Yusof et al. [5] and Banjade et al. [10]. The observed values of the surface dose were found to range between 2.29% and 2.34% for 6 MV photons. Similarly, the surface dose values for 10 MV photons were found to be 4.69% and 5.29%, respectively. The PDD values for the examined particleboard phantoms at a depth beyond $d_{max}$ indicates no significant difference with percentage difference within the limit of 0.09–0.16% for 6 MV, while for 10 MV photon, the variations in the depth beyond $d_{max}$ were found to range between 0.37–0.70%, which is consistent with those of water and solid water phantoms.

#### 3.3.3. PDD Curves for Gafchromic EBT3 Radiochromic Films for Photon Beams

**Figure 7:**
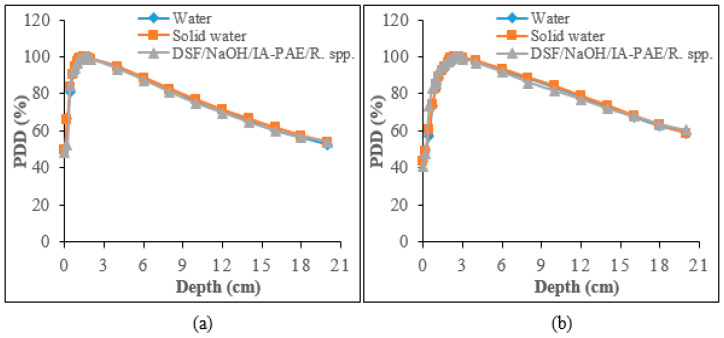
PDD plots with the use of EBT3 film for water, solid water, and DSF/NaOH/IA-PAE/*R.* spp. particleboard phantoms ascertained for: (**a**) 6 MV and (**b**) 10 MV photons.

Figure 7 shows a comparison between the estimated PDD profiles for sample A15, water, and solid water phantoms for 6 MV and 10 MV photon beams using Gafchromic EBT3 radiochromic films. As shown in the Figures, the percentage variations in the $d_{max}$ of the particleboard phantom, relative to those of water and solid water phantoms, were found to be 1.03% and 1.68% for 6 MV photons (Figure 7a), whereas the contrast between the measured PDD at all depths for 10 MV photon beams indicates good consistency with a difference within the range of 5.42% and 5.92% (Figure 7b). Overall, the results depict agreement with those of water and solid water phantoms in the build-up region for 6 MV photons with lower variations in the PDD values at $d_{max}$, whereas the variations were marginally higher for 10 MV photons with discrepancies found within 5% and 7%. This can be ascribed to the fact that the dominant free electron population originating in the build-up region continues to cause further interactions as a result of pair production, Compton scattering, and the photoelectric effect. High energy electrons are emitted as high energy photons (10 MV) interact with the phantoms and are absorbed by their interaction with the phantom. The resulting electrons will reduce with depth inside the phantoms owing to the continuously reduced energy fluence of the photons. The corresponding results for the surface dose of fabricated particleboards with water and solid water phantoms were found to be within 2.23–2.44% and 4.48–4.84%. With regards to the depth beyond $d_{max}$, the PDD values showed agreement to those of water and solid water phantoms, with percentage deviation in the interval of 0.01–0.02% and 0.06–0.07%, respectively. These trends are similar to what was reported for IC performance.

#### 3.3.4. Beam Profile Comparison at Reference Dose ($d_{ref}$) and Maximum Dose ($d_{max}$)

**Figure 8:**
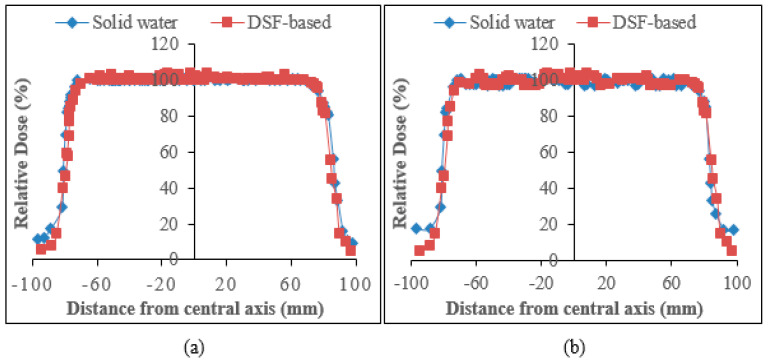
Beam profile for 6 MV photons evaluated at: (**a**) $d_{max}$ and (**b**) $d_{ref}$.

**Figure 9:**
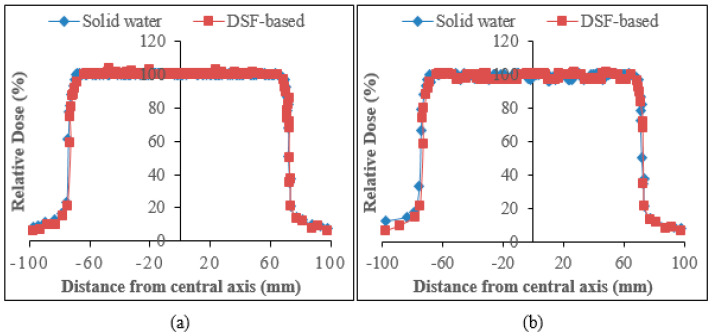
Beam profile for 10 MV photons measured at: (**a**) $d_{max}$ and (**b**) $d_{ref}$.

The comparison of the relative dose plots against distance from the central axis of sample A_15_ and solid water phantoms for the beam profile curves for 6 and 10 MV photons are presented in Figure 8 and Figure 9. As can be seen from the figures, the DSF-based particleboards reveal remarkable beam profiles with good dose homogeneity and beam symmetry in comparison to those of solid water phantoms. There was a consistency between the constructed particleboard and solid water phantom plots in both the dose plateau and the penumbra regions. Table 10 addressed the variation of flatness of the beam profiles at $d_{ref}$ and $d_{max}$ between the DSF-based particleboards and solid water phantoms for both photon energies. Overall, the beam uniformity enhanced as the photon energy increased, with 10 MV photons having a reduced percentage discrepancy of beam flatness values at $d_{ref}$ and $d_{max}$ compared with that of 6 MV photons relative to solid water phantom. This has demonstrated the appropriateness of DSF/NaOH/IA-PAE/*R.* spp. particleboards to be utilized as phantom material for high-energy photons in medical health applications.

#### 3.3.5. Determination of PDD for Electron Beams Using IC

**Figure 10:**
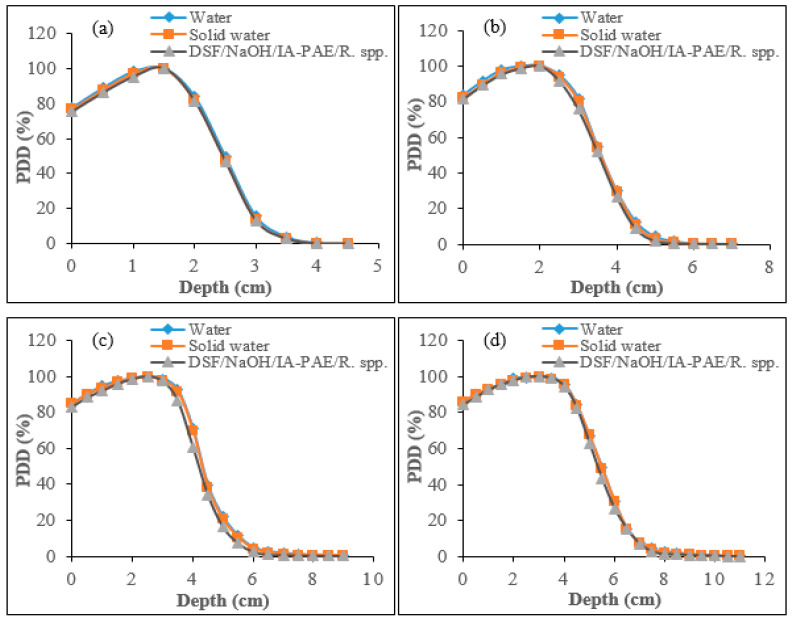
PDD curves for water, solid water, and DSF/NaOH/IA-PAE/*R.* spp. particleboard phantoms using IC for: (**a**) 6 MeV, (**b**) 9 MeV, (**c**) 12 MeV, and (**d**) 15 MeV electrons.

The PDD curves of the electron beams for the particleboard phantoms showed an improved surface dose when compared with that of water and solid water phantoms, as displayed in Figure 10a–d. DSF-based phantom delivers a reasonably homogeneous dose from the surface to a specific depth, after which the dose falls off rapidly with increasing depth, eventually to near zero values. As can be seen from the figures, the percentage dose variations in $d_{max}$ between the DSF-based *R.* spp. Particleboards, with respect to water and solid water phantoms for the four electron beam energies, were within 2.40–3.87%, 3.52–3.59%, 4.36–4.55%, and 2.82–4.63%, respectively. In addition, the percentage difference at which the electron PDD beyond the depth of $z_{max}$ drops off sharply as a result of the scattering and continuous energy loss by the incident electrons. The therapeutic range (R90 and R80) and half-value depth range (R50) were found to be within the limit and similar to those of water and solid water phantoms for 6, 9, 12, and 15 MeV electrons (Table 11).

#### 3.3.6. Evaluation of PDD for Electron Beams Using Gafchromic EBT3 Radiochromic Film

**Figure 11:**
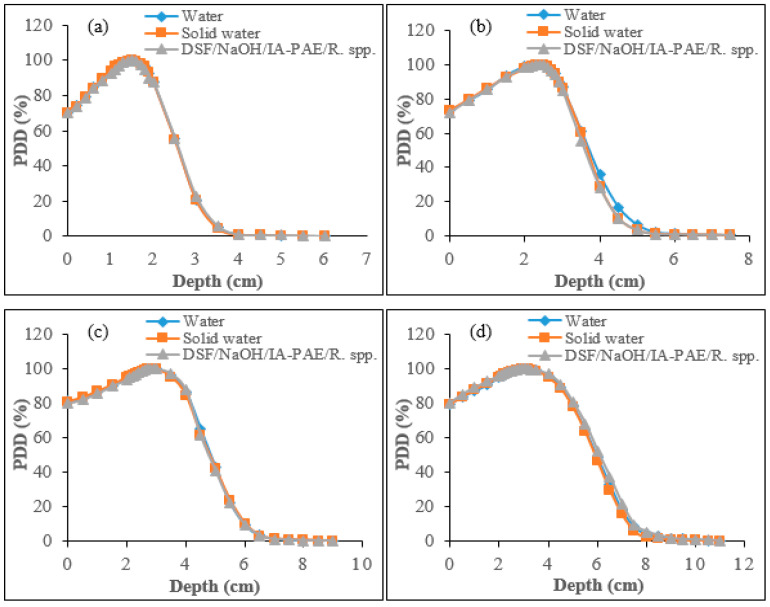
PDD plots for water, solid water, and DSF/NaOH/IA-PAE/*R.* spp. particleboard phantoms with the use of Gafchromic EBT3 radiochromic film for: (**a**) 6 MeV, (**b**) 9 MeV, (**c**) 12 MeV, and (**d**) 15 MeV electrons.

Figure 11a–d depicts the PDD profiles between DSF-based particleboard phantom, water, and solid water phantom evaluated from their surfaces for 6, 9, 12, and 15 MeV electron beams using Gafchromic EBT3 radiochromic films. As shown in the figures, comparable results were found in the constructed particleboards at the selected electron beams range to those of water and solid water phantoms. In this case, for 6, 9, 12, 15 MeV electrons discrepancies found were within 1.49–1.90%, 1.89–3.01%, 1.74–3.53%, and 2.38–3.84%, respectively. These findings indicate that, at 6 MeV, DSF-based particleboards depicted good agreement to those of water and solid water phantom with minimum discrepancies, whereas 9, 12, and 15 MeV give maximum values of percentage of discrepancies. Additionally, it can be observed that variations in percentage between the examined phantoms were lower at a depth beyond $z_{max}$ in comparison to that in the build-up region. According to the obtained results, the dissimilarities in the discrepancy of the surface dose values were found to improve in the range between 1.45–1.63%, 1.51–1.79%, 1.53–2.17%, and 1.98–2.70%, which showed good agreement with the results of the IC. The observed reduction in surface dose can be assigned to a slight reduction in backscatter. This confirms that EBT3 radiochromic film is suitable and provided surface dosimetry measurements in 6, 9, 12, and 15 MeV electrons beam fields.

## 4. Conclusions

The RAPs and dosimetric characterization of DSF/NaOH/*R.* spp. particleboard phantoms as a tissue-equivalent phantom material with different amounts of IA-PAE (0, 5, 10, and 15 wt%) have been demonstrated. The ascertained average mass density exhibited acceptable quality values to those of water and solid water phantom in the range between 0.99 ± 0.01 gcm^−3^–1.04 ± 0.03 gcm^−3^. The PMDSP, $z_{eff}$, and $N_{el}$ values were found to be satisfactory. Comparison between the calculated RAPs values shows a reasonable agreement with 15 wt% IA-PAE, solid water phantom, and theoretical values of water (XCOM), as indicated by the $\chi^{2}$ values (0.044). The dosimetric computation results of DSF/NaOH/IA-PAE/R. spp. particleboard phantoms from IC showed good agreement with Gafchromic EBT3 radiochromic films, and they were benchmarked with those of water and solid water phantoms for the selected high energy photons and electrons, demonstrating the possibility to use these dosimeters under extremely intense radiation fields and confirming the effectiveness of the DSF/NaOH/IA-PAE/*R.* spp. particleboards. The fabricated particleboard phantom (sample A_15_) was shown to be ideal for use in radiation therapy dosimetry as tissue-equivalent phantom material within the range of 1% variations to those of water and solid water phantoms.

## Figures and Tables

**Table 1:** PMDSP of particleboard phantoms.

Sample	Physico-Mechanical Property					Dimensional Stability Property			
	MC (%)	SC (%)	IB (MPa)	MOR (MPa)	MOE (GPa)	TS (%)		WA (%)	
						2 h	24 h	2 h	24 h
**A_0_**	**7.44 ± 0.21**	**33.18 ± 0.40**	**0.11 ± 0.09**	**5.39 ± 0.57**	2.04 ± 0.10	34.62 ± 1.8	50.3 ± 1.7	60.2 ± 1.1	66.1 ± 0.9
A_5_	8.08 ± 0.30	34.10 ± 1.08	0.65 ± 0.02	14.18 ± 0.31	4.46 ± 0.51	21.11 ± 1.0	25.2 ± 1.3	31.4 ± 1.6	36.3 ± 1.0
A_10_	7.11 ± 0.27	35.06 ± 0.56	0.69 ± 0.06	17.60 ± 0.45	7.30 ± 0.13	10.20 ± 1.2	11.0 ± 1.8	24.2 ± 0.8	30.6 ± 1.2
A_15_	7.05 ± 0.19	37.31 ± 1.01	0.72 ± 0.01	18.97 ± 0.22	7.89 ± 0.11	10.01 ± 0.9	10.5 ± 1.2	20.7 ± 1.0	23.9 ± 0.5

Remark: A_0_ = Uncured *R.* spp., A_5_ = DSF/NaOH/IA-PAE/*R.* spp., A_10_ = DSF/NaOH/IA-PAE/*R.* spp., and A_15_ = DSF/NaOH/IA-PAE/*R.* spp. indicate 0, 5, 10, and 15 wt% IA-PAE. Data are expressed as an average ± standard deviation (SD).

**Table 2:** Elemental compositions, $Z_{eff}$, and $N_{el}$ of phantom samples.

Sample	Weight Fraction of Elements in Each Sample (%)													$Z_{eff}$	$N_{el}\times 10^{23}$
	H	C	O	N	Na	Mg	*p*	S	Cl	K	Ca	Fe	Zn		
A_0_	-	51.01	46.24	2.64	-	-	-	0.11	-	-	-	-		7.18 ^a^	3.39
A_5_	-	51.48	42.10	4.71	0.55	0.20	-	-	0.15	0.21	0.60	-	-	7.45 ^a^	3.26
A_10_	-	51.07	43.92	4.02	0.28	-	-	-	0.11	0.24	0.31	0.05		7.51 ^a^	3.33
A_15_	-	53.11	41.28	2.56	0.43	0.04	0.12	0.05	0.32	0.18	1.75	0.07	0.09	7.72 ^a^	3.34
Solid water	8.10	67.20	19.90	2.40	-	-	-	-	0.10	-	2.30	-		7.29 ^b^	3.32
Water	11.20	-	88.80	-	-	-	-	-	-	-	-	-		7.42 ^b^	3.34
Virtual water	7.70	68.70	18.90	2.30	-	-	-	-	0.10	-	2.30	-	-	6.12 ^c^	3.38
PMMA	8.00	60.00	31.96	-	-	-	-	-	-	-	-	-	-	5.85 ^c^	3.87
Polystyrene	7.74	92.26	-	-	-	-	-	-	-	-	-	-	-	5.29 ^c^	3.43

^a^ Curent study, ^b^ Sahoo et al. [27], ^c^ Schoenfeld et al. [28].

**Table 3:** Parameters of various standard phantom materials compared with DSF/NaOH/IA-PAE/*R.* spp.

Phantom Materials	Manufacturer	Density (g/cm^3^)	Mean HU Value	ED × 10^23^ (Electrons/cm^3^)
A_0_	Current study	1.07	−89.71	3.39 ^a^
A_5_	Current study	0.96	−55.55	3.26 ^a^
A_10_	Current study	0.99	−33.01	3.33 ^a^
A_15_	Current study	1.01	−12.79	3.34 ^a^
Solid water	Gammex, Middleton, WI, USA	1.04	5.30	3.32 ^b^
Water	-	1.00	−9.01	3.34 ^b^
Virtual water	Med-Cal, Middleton, WI, USA	1.04	−7 ± 7	3.38 ^c^
Polymethyl methacrylate	-	1.19	133	3.87 ^c^
Polystyrene	-	1.06	140.5	3.43 ^c^

^a^ Current study, ^b^ Sahoo et al. [27], ^c^ Schoenfeld et al. [28].

**Table 4:** LAC and MAC values of DSF/NaOH/IA-PAE/*R.* spp. particleboards and solid water phantoms in comparison with water (XCOM).

Sample	Average ρ (gcm^−3^)	^137^Cs (0.662 MeV)			^60^Co (1.250 MeV)		
		μ (cm^−1^)	μ/ρ (cm^2^/g)	σ_μ/ρ_ ± (%)	μ (cm^−1^)	μ/ρ (cm^2^/g)	σ_μ/ρ_ ± (%)
A_0_	1.040	0.063	0.061	0.028	0.043	0.041	0.023
A_5_	1.038	0.067	0.064	0.021	0.045	0.044	0.017
A_10_	1.002	0.070	0.070	0.019	0.054	0.054	0.015
A_15_	1.006	0.083	0.082	0.009	0.056	0.056	0.011
Solid water	1.040	0.085	0.082	0.013	0.058	0.056	0.017
Water (XCOM)	1.000	0.086			0.059		

**Table 5:** $\chi^{2}$ values for MAC of DSF-based *R.* spp. particleboards and solid water phantoms.

Sample	$\chi^{2}$ Water (XCOM)	
	^137^Cs (0.662 MeV)	^60^Co (1.250 MeV)
A_0_	0.797	0.612
A_5_	1.098	0.779
A_10_	0.709	0.111
A_15_	0.198	0.044
Solid water	0.095	0.031

**Table 6:** TPR_20,10_ measurement for DSF/NaOH/IA-PAE/*R.* spp. particleboards (sample A_15_), water, and solid water phantoms for 6 MV photons.

Phantom	Depth (cm)	Charge Collected (nC)			Mean	Ratio	Discrepancy (%)		
		1	2	3			W/S	W/*R*	S/*R*
Water	20	9.786	9.791	9.792	9.789	0.698	-	-	-
	10	14.04	14.01	14.03	14.027				
Solid water	20	9.511	9.509	9.512	9.511	0.695	0.43	-	-
	10	13.69	13.69	13.69	13.690				
DSF-based	20	9.845	9.836	9.841	9.841	0.693	-	0.72	0.29
	10	14.22	14.20	14.20	14.207				

Note: W, S, and *R* depict the water, solid water, and DSF-based (DSF/NaOH/IA-PAE/*R.* spp.) phantoms.

**Table 7:** TPR_20,10_ evaluation for DSF/NaOH/IA-PAE/*R.* spp. particleboards (sample A_15_), water, and solid water phantoms for 10 MV photons.

Phantom	Depth (cm)	Charge Collected (nC)			Mean	Ratio	Discrepancy (%)		
		1	2	3			W/S	W/*R*	S/*R*
Water	20	12.23	12.20	12.22	12.217	0.770	-	-	-
	10	15.86	15.86	15.86	15.860				
Solid water	20	12.05	12.08	12.08	12.070	0.767	0.39	-	-
	10	15.74	15.73	15.73	15.733				
DSF-based	20	12.32	12.31	12.31	12.313	0.765	-	0.65	0.26
	10	16.10	16.10	16.09	16.097				

**Table 8:** Paired *t*-test of the TPR_20,10_ measurement for 6 MV photons.

Phantom	Paired Differences					*t*	df	Sig. (2-Tailed)
	Mean (d)	Std. Dev. $\left(\sigma_{d}\right)$	Std. Error Mean	95% Confidence Interval Difference				
				Lower	Upper			
Water	0.698	0.0082	0.0004	0.6967	0.6993	1709.7	20	0.081
Solid water	0.695	0.0016	0.0082	0.6924	0.6976	851.2	20	0.073
DSF-based	0.693	0.0022	0.0011	0.6896	0.6964	641.59	20	0.071

**Table 9:** Paired *t*-test of the TPR_20,10_ measurement for 10 MV photons.

Phantom	Paired Differences					*t*	df	Sig. (2-Tailed)
	Mean (d)	Std. Dev. $\left(\sigma_{d}\right)$	Std. Error Mean	95% Confidence Interval Difference				
				Lower	Upper			
Water	0.770	0.0008	0.0004	0.7690	0.7715	1942.2	20	0.086
Solid water	0.767	0.0011	0.0005	0.7654	0.7689	1414.4	20	0.077
DSF-based	0.765	0.0010	0.0005	0.7638	0.7670	1486.8	20	0.069

**Table 10:** Beam profile flatness for DSF/NaOH/IA-PAE/*R.* spp. particleboards (sample A_15_) compared to that of solid water phantom for 6 MV and 10 MV photons.

Phantom	Beam Flatness				Discrepancy (%)			
	6 MV		10 MV		6 MV		10 MV	
	5 cm	$z_{max}$	5 cm	$z_{max}$	5 cm	$z_{max}$	5 cm	$z_{max}$
Solid water	2.582	2.416	1.933	1.816	-	-	-	-
DSF-based	2.761	2.596	2.010	1.923	6.93	7.45	3.98	5.89

**Table 11:** Comparison of PDD curves between DSF/NaOH/IA-PAE/*R.* spp., water and solid water phantoms for different electron beams.

Depth	Percentage Difference of PDD (%)							
	Water				Solid Water			
	6 MeV	9 MeV	12 MeV	15 MeV	6 MeV	9 MeV	12 MeV	15 MeV
$z_{max}$	2.06	1.33	1.27	1.81	1.51	0.79	1.01	1.30
d_50_	2.34	2.75	2.73	2.22	1.69	1.56	1.09	2.02
d_80_	2.69	2.54	2.84	2.05	1.71	2.33	1.34	2.67
d_90_	2.51	2.78	2.64	2.40	1.60	2.89	1.78	2.44

## Data Availability

Not applicable.
